# Machine Learning–Based Prediction of Early Complications Following Surgery for Intestinal Obstruction: Multicenter Retrospective Study

**DOI:** 10.2196/68354

**Published:** 2025-03-03

**Authors:** Pinjie Huang, Jirong Yang, Dizhou Zhao, Taojia Ran, Yuheng Luo, Dong Yang, Xueqin Zheng, Shaoli Zhou, Chaojin Chen

**Affiliations:** 1 Department of Anesthesiology Third Affiliated Hospital of Sun Yat-sen University Guangzhou China; 2 Department of Anesthesiology ShenZhen People’s Hospital Shenzhen China; 3 Guangzhou AI & Data Cloud Technology Co. Guangzhou China; 4 Department of Anesthesiology First People's Hospital of Foshan Foshan China

**Keywords:** postoperative complications, intestinal obstruction, machine learning, early intervention, risk calculator, prediction model, Shapley additive explanations

## Abstract

**Background:**

Early complications increase in-hospital stay and mortality after intestinal obstruction surgery. It is important to identify the risk of postoperative early complications for patients with intestinal obstruction at a sufficiently early stage, which would allow preemptive individualized enhanced therapy to be conducted to improve the prognosis of patients with intestinal obstruction. A risk predictive model based on machine learning is helpful for early diagnosis and timely intervention.

**Objective:**

This study aimed to construct an online risk calculator for early postoperative complications in patients after intestinal obstruction surgery based on machine learning algorithms.

**Methods:**

A total of 396 patients undergoing intestinal obstruction surgery from April 2013 to April 2021 at an independent medical center were enrolled as the training cohort. Overall, 7 machine learning methods were used to establish prediction models, with their performance appraised via the area under the receiver operating characteristic curve (AUROC), accuracy, sensitivity, specificity, and *F*_1_-score. The best model was validated through 2 independent medical centers, a publicly available perioperative dataset the Informative Surgical Patient dataset for Innovative Research Environment (INSPIRE), and a mixed cohort consisting of the above 3 datasets, involving 50, 66, 48, and 164 cases, respectively. Shapley Additive Explanations were measured to identify risk factors.

**Results:**

The incidence of postoperative complications in the training cohort was 47.44% (176/371), while the incidences in 4 external validation cohorts were 34% (17/50), 56.06% (37/66), 52.08% (25/48), and 48.17% (79/164), respectively. Postoperative complications were associated with 8-item features: Physiological Severity Score for the Enumeration of Mortality and Morbidity (POSSUM physiological score), the amount of colloid infusion, shock index before anesthesia induction, ASA (American Society of Anesthesiologists) classification, the percentage of neutrophils, shock index at the end of surgery, age, and total protein. The random forest model showed the best overall performance, with an AUROC of 0.788 (95% CI 0.709-0.869), accuracy of 0.756, sensitivity of 0.695, specificity of 0.810, and *F*_1_-score of 0.727 in the training cohort. The random forest model also achieved a comparable AUROC of 0.755 (95% CI 0.652-0.839) in validation cohort 1, a greater AUROC of 0.817 (95% CI 0.695-0.913) in validation cohort 2, a similar AUROC of 0.786 (95% CI 0.628-0.902) in validation cohort 3, and the comparable AUROC of 0.720 (95% CI 0.671-0.768) in validation cohort 4. We visualized the random forest model and created a web-based online risk calculator.

**Conclusions:**

We have developed and validated a generalizable random forest model to predict postoperative early complications in patients undergoing intestinal obstruction surgery, enabling clinicians to screen high-risk patients and implement early individualized interventions. An online risk calculator for early postoperative complications was developed to make the random forest model accessible to clinicians around the world.

## Introduction

Early postoperative complications refer to newly occurring situations or events that are detrimental to the patient’s health from the first postoperative day until discharge, causing irreversible damage or requiring a change in treatment policy [1]. The incidence of early postoperative complications in intestinal obstruction was reported to be as high as 23%-28%, which contributed to an increased length of hospital stay and mortality [2,3]. Early complications after intestinal obstruction surgery include postoperative infection, wound dehiscence, important organs’ dysfunction, intestinal fistula, postoperative bleeding, and other surgery-related complications [3,4]. Surgery is a reliable means for relieving obstruction, but for frail individuals, surgery, anesthesia, and various perioperative interventions pose challenges as well. Accurate prediction of early postoperative complications is essential for proper preoperative selection of surgical patients, determination of the necessary postoperative vigilance level, guidance of perioperative decision-making, and early intervention.

Currently, studies have found that delayed surgery, American Society of Anesthesiologists (ASA) physical status classification, age, and surgical methods may be risk factors for its occurrence [4-6]. A study proposed using the Acute General Emergency Surgical Severity–Small Bowel Obstruction (AGESS-SBO) score [7] to predict the prognosis of small-bowel obstruction (SBO), but the current analysis and early intervention still depend on expert consensus and guidelines. A predictive model for identifying high-risk patients is required to optimize the treatment strategy for intestinal obstruction. However, the intricate etiology, pathophysiology, and perioperative changes of intestinal obstruction render traditional logistic regression models not applicable.

Data-driven machine learning (ML) modeling, a technology used to build data-driven artificial intelligence systems, has the ability to diagnose and predict the prognosis of patients. On the one hand, ML has the advantages of capturing nonlinear relationships more comprehensively and predicting the prognosis accurately [8]; on the other hand, with the increasing complexity and dimensions of modern medical datasets, ML can give full play to the advantage of a rich data volume in big data. These factors make it popular in medicine, including anesthesiology [9], cardiology [10], ophthalmology [11], and so on. ML has been used to predict postoperative complications. Some cohort studies have applied ML to diagnose intestinal obstruction [12], but it has not been applied to predict early postoperative complications. While the performance of ML models is superior to traditional scoring systems, the limitation of these models is that they are mostly designed on the basis of single-center data. What is important is that the ML prediction models have not been transformed into practical applications.

In this multicenter retrospective study, we developed an online risk calculator for early postoperative complications of patients with intestinal obstruction in an independent medical center and performed external validation through two independent medical centers, a publicly available perioperative dataset Informative Surgical Patient dataset for Innovative Research Environment (INSPIRE) and a mixed cohort consisting of the above 3 external validation datasets.

## Methods

### Source of the Data and Participants

This retrospective study was performed using a multicenter database of patients who underwent surgery for intestinal obstruction: the Third Affiliated Hospital of Sun Yat-sen University, Shenzhen People’s Hospital, Foshan First People’s Hospital, and the INSPIRE dataset. INSPIRE is a publicly available research dataset for perioperative medicine, which includes approximately 130,000 patients (50% of all surgical patients) who underwent anesthesia for surgery at an academic institution in South Korea between 2011 and 2020 [13,14]. To our knowledge, it is a new dataset that contains data for collaborative research and development in perioperative medicine.

The medical data collected from electronic medical record systems and perioperative databases included demographic data, preoperative comorbidities, laboratory results, drugs, intraoperative data, and diagnosis-related features.

**Figure 1:**
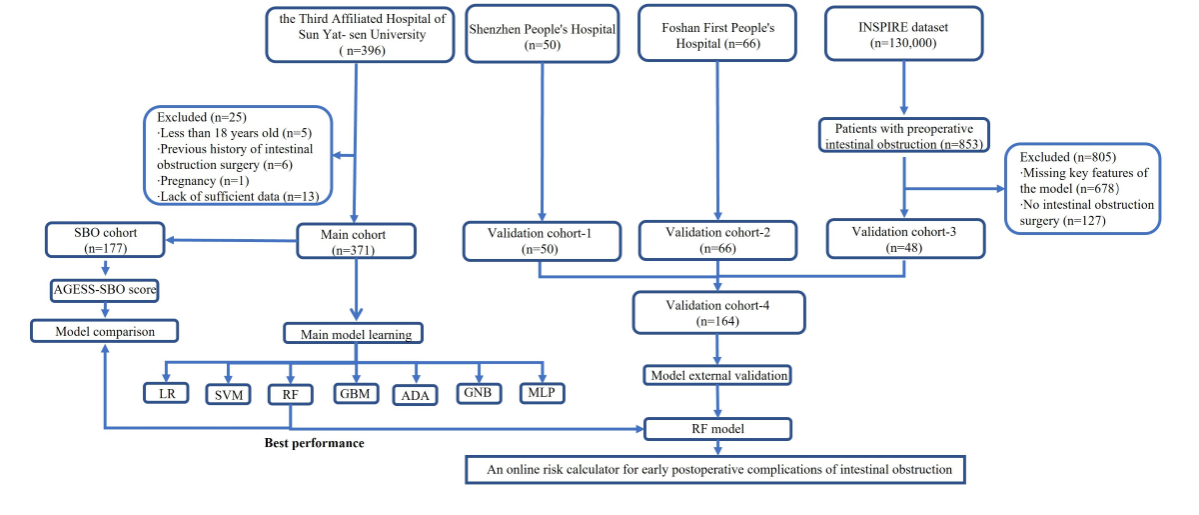
Flowchart of patient enrollment. ADA: adaptive boosting classifier; GBM: gradient boosting machine; GNB: Gaussian Bayesian classifier; LR: logistic regression; MLP: multilayer perceptron; RF: random forest; SVM: support vector machine.

As a result, 396 patients who underwent surgery for intestinal obstruction from April 2013 to April 2021 were extracted. After excluding minors, pregnant women, patients who underwent secondary procedures for intestinal obstruction, and patients who lacked sufficient related records, 371 patients from the Third Affiliated Hospital of Sun Yat-sen University were included in the primary cohort for model development. A total of 116 patients who underwent surgery for intestinal obstruction meeting the same inclusion criteria from Shenzhen People’s Hospital (50 patients, 2021-2022) and Foshan First People’s Hospital (66 patients, 2020-2022) were exclusively extracted for the validation cohorts 1 and 2. We retrieved 853 patients with preoperative intestinal obstruction from the INSPIRE database of 130,000 patients. After excluding patients without key variables required for the model (678 patients) and nonintestinal obstruction surgical patients (127 patients), the remaining 48 patients were selected as validation cohort 3. Finally, the patients from 3 external validation cohorts jointly formed the external validation cohort 4 (Figure 1).

### Model Outcome

The primary outcome was to assess early postoperative complications occurring from the first postoperative day until discharge. According to the definition provided by the Association of Surgeons of the Netherlands (ASN), we defined these complications as “a condition or an event that is detrimental to the patient’s health, causing irreversible damage or requiring a change in treatment policy.” The complications were identified using the *ICD-10* (*International Statistical Classification of Diseases and Related Health Problems 10th Revision*) diagnosis codes and only those labeled as “not present on admission” on the medical record were counted as postoperative complications to ensure that events within the composite outcome were newly developed. We classified these complications in accordance with the Clavien-Dindo [15] (Table S1 in Multimedia Appendix 1) classification and the patients’ treatment strategies, to provide more information. Predictions of all levels of complications will be made. Specifically, sepsis was defined as a life-threatening organ dysfunction caused by the host’s maladjusted response to infection. The diagnosis of sepsis is based on Sepsis 3.0 diagnostic criteria. Infections or suspected infections include lung infections, intestinal infections, abdominal infections, urinary system infections, and so on, and were manifested as positive pathogen culture or symptoms of infection, but without organ dysfunction. Poor wound healing included wound infection, fat liquefaction, and nonepithelial regeneration of the wound surface accompanied by hematoma, wound dehiscence, and excessive scar hyperplasia.

### Predictors, Missing Data, and Selection

**Figure 2:**
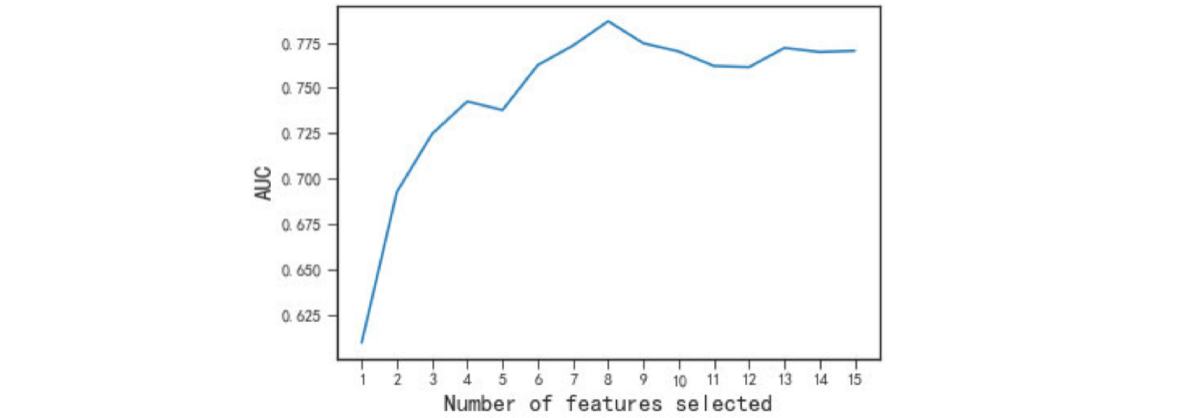
The process of using a wrapper feature selection method to screen features. Upon the initial selection of the first 8 features, the model achieved its peak area under the receiver operating characteristic (AUROC) curve on the validation set. Subsequent addition of features did not result in a further increase of the AUROC. Consequently, we opted for the top 8 features with the highest Shapley Additive Explanations value.

Variables with more than 30% missing data and patients with more than 50% missing variables were excluded from further analysis. After doing this, a total of 127 variables were chosen for the univariate analysis, mainly covering demographic characteristics, preoperative comorbidities, laboratory values, cause and complications of intestinal obstruction, as well as intraoperative incidents, shock index (SI), medication, and fluid infusion (Table S2 in Multimedia Appendix 1). Some classification variables were produced by imposing specific rules based on their definitions. According to the statistical analysis of data correlation, only features that had statistical significance (*P*<.05) in the univariate test were selected to minimize the potential overfitting caused by the high dimensions of features. A total of 71 variables were chosen, and then the missing values of these 71 variables were filled. The mode-filling method was used for categorical indicators, and the K-nearest neighbor algorithm was used for continuous indicators. After conducting univariate analysis, we next performed the least absolute shrinkage and selection operator (LASSO) regression analysis on the residual variables. Subsequently, we eliminated variables with LASSO coefficients below 0.02, resulting in the selection of 14 variables. On this basis, we used the wrapper method based on the model score to screen features again. We trained a support vector machine model with 14 variables to calculate their Shapley Additive Explanations (SHAP) values using the training set. We then sorted the variables in descending order according to their SHAP values. These variables were then successively added to the support vector machine model for retraining while monitoring the area under the receiver operating characteristic curve (AUROC) of the model on the validation set. Ultimately, upon selecting the initial 8 indicators, the model’s AUROC on the training set reached its maximum (Figure 2).

### Statistical Analysis

Python (version 3.9.12) was used as the base platform. Table processing was conducted using Pandas (version 1.3.5). The SciPy package (version 1.7.1) was used to analyze the data. The Sklearn software package (version 1.1.2) was used for data preprocessing and building the base models, including logistic regression, support vector machine, random forest (RF), gradient boosting machine implemented by a decision tree, adaptive boosting, the Gaussian Bayesian classifier, and multilayer perceptron.

The main cohort was randomly divided into a 70% development set and a 30% internal validation set. The bootstrap method was used to perform 1000 runs on the internal validation set to calculate confidence intervals for AUROC, accuracy, sensitivity, and specificity. Continuous variables were analyzed and expressed as median values with interquartile intervals and compared using the independent-sample *t* test or Mann-Whitney *U* test. Categorical variables are expressed in terms of quantity and percentage and were compared using a chi-square test. The SHAP method was implemented using the Python SHAP package.

### Model Validation

The best-performing model was compared with the AGESS-SBO score, which is calculated by the American Association for the Surgery of Trauma (AAST) anatomical score, physiology score, and comorbidity score as the basis for risk stratification [7]. The ML model was also compared and validated on the data from two other independent cohorts, the INSPIRE dataset, and a mixed cohort to demonstrate its extrapolation and generalization. The prediction performances were compared in terms of AUROC, specificity, sensitivity, accuracy, and *F*_1_-score.

### Ethical Considerations

The study protocol followed the principles of the Declaration of Helsinki and was approved by the Institutional Ethics Committee of the Third Affiliated Hospital of Sun Yat-sen University on October 19, 2022 ([2022] 02-004-02). Study data were anonymous or deidentified. The requirements for informed consent and clinical trial registration were waived by the committee. No identification of individual participants in any images of the manuscript or supplementary material was possible.

## Results

### Univariate Analysis

Among the 396 patients undergoing intestinal obstruction surgery accessed from the EHRs, only 371 patients who met the inclusion criteria were included in the development cohort. The development cohort consisted of a majority of males (242/371, 65.22%), with a median age of 61 (IQR 47-70) years and a median BMI of 20.96 (IQR 19.03-23.81) kg/m^2^. The flowchart of the study design is shown in Figure 1. Among the 371 patients included, 176 (47.44%) were diagnosed with early postoperative complications (complication group). The first 3 common postoperative complications were infection (26.95%), sepsis (16.17%), and poor wound healing (7.82%, Table 1). According to Clavien-Dindo’s classification of complications [15], grade I had the highest incidence (15.63%, Table 1).

**Table 1:** The incidence of the 12 most severe and common postoperative complications in the development cohort and the classification of patients in the development cohort based on the Clavien-Dindo classification.

Postoperative complications^a^		Values (N=371), n (%)
Death		16 (4.31)
Sepsis		60 (16.17)
Infection or suspected infection		100 (26.95)
Heart failure		17 (4.58)
Respiratory failure		26 (7.01)
Pulmonary embolism		2 (0.54)
Acute hepatic insufficiency		12 (3.23)
Acute kidney injury		10 (2.7)
Gastrointestinal bleeding		21 (5.66)
Recurrent intestinal obstruction		20 (5.39)
Anastomotic fistula		8 (2.16)
Poor wound healing		29 (7.82)
**Clavien-Dindo classification**		
	Grade I	58 (15.63)
	Grade II	55 (14.82)
	Grade IIIa	8 (2.16)
	Grade IIIb	12 (3.23)
	Grade Iva	13 (3.5)
	Grade IVb	14 (3.77)
	Grade V	16 (4.31)

^a^Some severe and common complications are listed here.

A total of 127 perioperative variables were analyzed by univariate analysis. Finally, only 71 variables with significant differences (*P*<.05) between the two groups were retained, including 37 preoperative variables and 34 intraoperative variables (Table S2 in Multimedia Appendix 1).

Compared with the patients without early postoperative complications, the time of first postoperative defecation, time of drainage tube retention, total hospitalization cost, total hospitalization days, postoperative hospitalization days, and ICU (intensive care unit) length of stay in the complication group was increased significantly (*P*<.05, Table S2 in Multimedia Appendix 1).

### Internal Validation Performance

After the univariate analysis, the LASSO regression analysis, and the wrapper method based on SHAP, we selected 8 features, including the Physiological Severity Score for the Enumeration of Mortality and Morbidity (POSSUM physiological score), the amount of colloid infusion, SI before anesthesia induction, the ASA classification, the percentage of neutrophils, SI at the end of the surgery, age, and total protein to train our 7 models. To increase the robustness of the model prediction results during the training process, we used the integration idea for modeling [16] and finally established 7 common ML models. In the training set, the RF model achieved a relatively balanced AUROC (0.788, 95% CI 0.709-0.869) and specificity (0.810, 95% CI 0.715-0.884) and the highest sensitivity (0.695, 95% CI 0.570-0.811), accuracy (0.756, 95% CI 0.696-0.831) and *F*_1_-score (0.727, 95% CI 0.633-0.812). Since GNB reached the greatest AUROC (0.805, 95% CI 0.730-0.878), its sensitivity was lowest (0.544, 95% CI 0.433-0.674). We eventually chose the RF model for further analysis and application.

**Figure 3:**
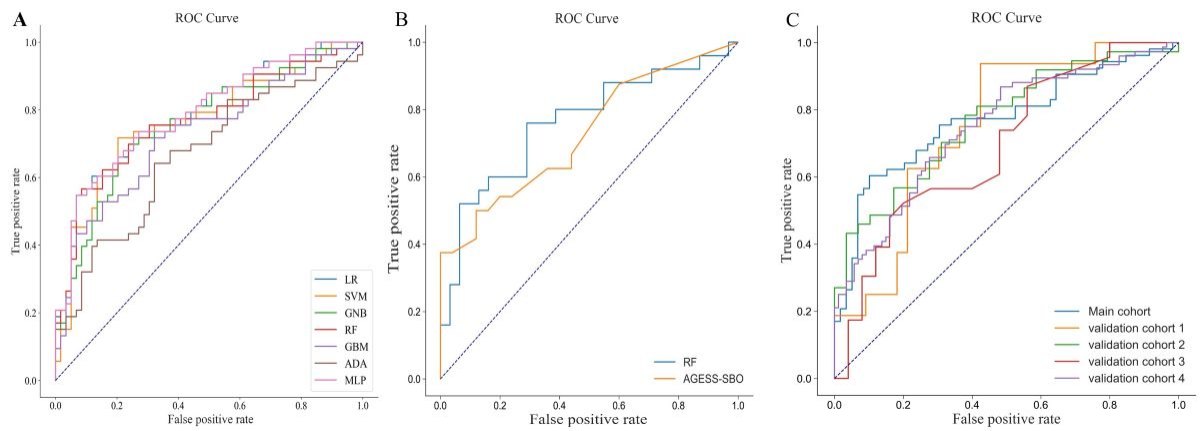
Use of the receiver operating characteristic curve plots to demonstrate the performance of the machine learning model in different cohorts. ADA: adaptive boosting; GBM: gradient boosting machine; GNB: Gaussian Bayesian classifier; LR: logistic regression; MLP: multilayer perceptron; RF: random forest; SVM: support vector machine.

Since the AGESS-SBO score is a scoring system for postoperative complications of SBO, we excluded patients with colorectal obstruction. Then, we validated and compared the performance of this score and ML predictor in a set excluding patients with colorectal obstruction. The AGESS-SBO score presented a higher specificity (0.889, 95% CI 0.750-1.0) with a lower AUROC (0.731, 95% CI 0.585-0.854), accuracy (0.653, 95% CI 0.531-0.776), *F*_1_-score (0.537, 95% CI 0.320-0.718), and sensitivity (0.409, 95% CI 0.222-0.625). For predicting early complications of SBO, the RF model achieved the highest AUROC (0.912, 95% CI 0.809-0.986), accuracy (0.831, 95% CI 0.735-0.918), sensitivity (0.893, 95% CI 0.725-1.0), *F*_1_-score (0.817, 95% CI 0.684-0.929), and balanced specificity (0.786, 95% CI 0.619-0.93; Table 2, Figures 3A and 3B).

**Table 2:** The performance of machine learning models in training sets and external validation cohorts.

Algorithm	AUROC^a^ (95% CI)	Accuracy (95% CI)	Sensitivity (95% CI)	Specificity (95% CI)	*F*_1_-score (95% CI)
LR^b^	0.797 (0.693-0.881)	0.733 (0.661-0.812)	0.695 (0.565-0.814)	0.769 (0.637-0.877)	0.713 (0.609-0.807)
SVM^c^	0.780 (0.690-0.854)	0.714 (0.638-0.781)	0.615 (0.504-0.731)	0.803 (0.680-0.889)	0.666 (0.570-0.756)
GNB^d^	0.805 (0.730-0.878)	0.698 (0.625-0.777)	0.544 (0.433-0.674)	0.833 (0.743-0.921)	0.625 (0.522-0.723)
RF^e^	0.788 (0.709-0.869)	0.756 (0.696-0.831)	0.695 (0.570-0.811)	0.810 (0.715-0.884)	0.727 (0.633-0.812)
GBM^f^	0.747 (0.667-0.836)	0.683 (0.598-0.777)	0.671 (0.570-0.794)	0.695 (0.568-0.831)	0.666 (0.567-0.774)
ADA^g^	0.668 (0.562-0.745)	0.665 (0.589-0.737)	0.688 (0.571-0.802)	0.644 (0.532-0.759)	0.655 (0.545-0.740)
MLP^h^	0.790 (0.708-0.862)	0.692 (0.598-0.759)	0.611 (0.454-0.724)	0.765 (0.652-0.870)	0.649 (0.520-0.733)
Validation cohort 1	0.755 (0.652-0.839)	0.729 (0.644-0.805)	0.621 (0.444-0.774)	0.772 (0.686-0.859)	0.568 (0.417-0.684)
Validation cohort 2	0.817 (0.695-0.913)	0.742 (0.636-0.848)	0.703 (0.553-0.845)	0.800 (0.625-0.936)	0.754 (0.620-0.853)
Validation cohort 3	0.786 (0.628- 0.902)	0.708 (0.562- 0.833)	0.700 (0.500- 0.870)	0.724 (0.522- 0.875)	0.700(0.529- 0.826)
Validation cohort 4	0.720 (0.671- 0.768)	0.658 (0.602- 0.702)	0.634 (0.566- 0.695)	0.679 (0.617- 0.730)	0.628 (0.575- 0.682)
RF-SBO^i^	0.912 (0.809-0.986)	0.831 (0.735-0.918)	0.893 (0.725-1.0)	0.786 (0.619-0.93)	0.817 (0.684-0.929)
AGESS-SBO^j^	0.731 (0.585-0.854)	0.653 (0.531-0.776)	0.409 (0.222-0.625)	0.889 (0.750-1.0)	0.537 (0.320-0.718)

^a^AUROC: area under the receiver operating characteristic curve.

^b^LR: logistic regression.

^c^SVM: support vector machine.

^d^GNB: Gaussian Bayesian classifier.

^e^RF: random forest.

^f^GBM: gradient boosting machine.

^g^ADA: adaptive boosting.

^h^MLP: multilayer perceptron.

^i^RF-SBO: random forest–small-bowel obstruction.

^j^AGESS-SBO: Acute General Emergency Surgical Severity-Small Bowel Obstruction.

**Figure 4:**
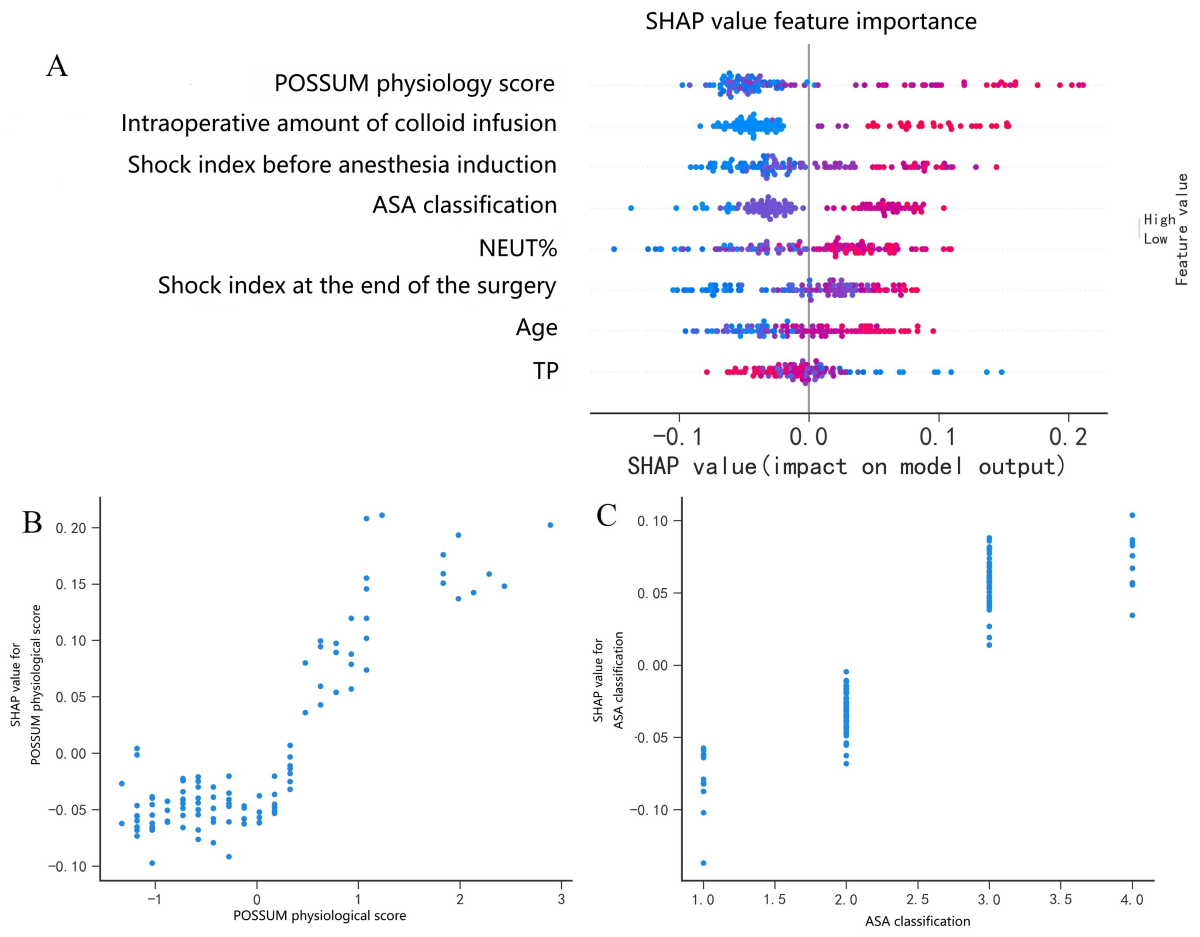
Use of Shapley Additive Explanations (SHAP) summary plot and SHAP dependence plot to demonstrate the importance and impact of variables on the random forest model. ASA: American Society of Anesthesiologists; SHAP: Shapley Additive Explanations; NEUT%: neutrophil percentage; POSSUM: Physiological and Operative Severity Score for the Enumeration of Mortality and Morbidity.

In Figure 3A, the receiver operating characteristic (ROC) curve of all machine learning models in the development set is shown. In Figure 4B, the ROC curve of the random forest model and AGESS-SBO score in the SBO cases of the development set is shown. In Figure 4C, the ROC curve of the random forest model in the validation sets is shown.

### External Validation Performance

The external validation cohorts included 50 patients from Shenzhen People’s Hospital (validation cohort 1), 66 patients from Foshan First People’s Hospital (validation cohort 2), 48 patients from the INSPIRE dataset (validation cohort 3), and 164 patients from the above 3 datasets (validation cohort 4). In these 4 external validation sets, the incidence of early postoperative complications was 34%, 56.06%, 52.08%, and 48.17%, respectively. In the four validation sets, the RF model achieved a comparable AUROC (0.755, 95% CI 0.652-0.839), a greater AUROC (0.817, 95% CI 0.695-0.913), a similar AUROC (0.786, 95% CI 0.628-0.902) and a comparable AUROC (0.720, 95% CI 0.671-0.768; Table 2 and Figure 3C).

### Feature Importance Evaluated by SHAP Values

The baseline for the SHAP value in this study is the average of all predicted early complication incidences in the internal validation set, which was 49.96%. Figure 4A shows the explanation of the ML model by SHAP. The SHAP summary plot demonstrated that the POSSUM physiological score, the amount of colloid infusion, SI before anesthesia induction, the ASA classification, the percentage of neutrophils, SI at the end of the surgery, age, and total protein were ranked as the top 8 important variables for RF. Both kinds of SHAP plots show that a higher POSSUM physiological score, larger amount of colloid infusion, larger SI before anesthesia induction, higher ASA grade, higher percentage of neutrophils, larger SI at the end of the surgery, older age, and lower total protein were associated with a higher SHAP value output in the RF model, indicating higher odds of early postoperative complications after surgery for intestinal obstruction.

We use a SHAP dependence plot to observe how a feature affects the prediction results of the model (Figure S1 in Multimedia Appendix 1; Figures 4B and 4C). For example, when the POSSUM physiological score is high, the SHAP value will change significantly, showing a positive correlation (Figure 4B). From the SHAP dependence plot, it can also be found that there is an obvious “truncation” in the SHAP value between ASA II and ASA III, indicating that ASA II and ASA III have a greater contribution to distinguishing the positive and negative samples (Figure 4C).

**Figure 5:**
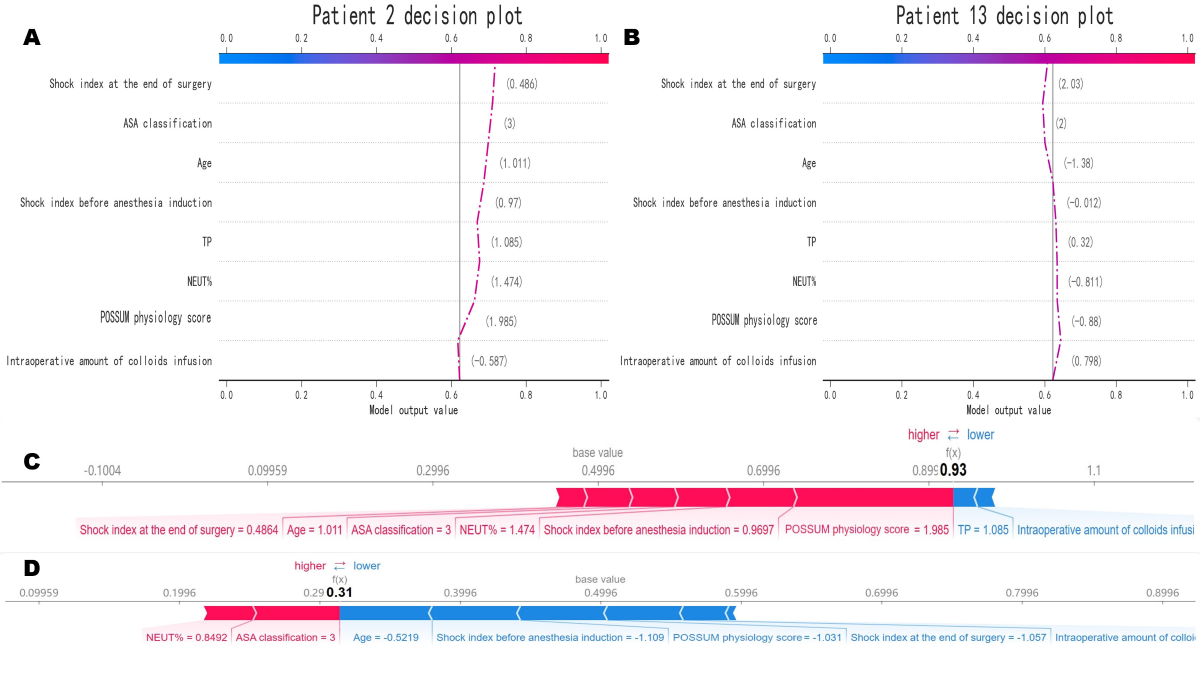
Use the SHAP decision plots and SHAP force plots to demonstrate the decision pathways and feature importance of the RF model in specific patients.

An example of correctly classified cases and an example of incorrectly classified cases were demonstrated as a SHAP decision plot (Figures 5A and 5B) and a force plot (Figures 5C and 5D). These plots increase the interpretability and transparency of the predictions made by RF algorithms. The SHAP decision plots showed how the model makes decisions based on the availability of each feature in the electronic medical record and provided a decision path for each feature. The force plot mainly shows the major factors that contribute to the final model output of a specific individual.

In Figure 4A, the SHAP summary plot illustrates the overall impact distribution of each feature on the RF model. The features are sorted on the basis of the average absolute value of their SHAP values, with the importance of the features decreasing from the top to the bottom. The color scheme can aid in understanding how changes in feature values affect the results. Red indicates high eigenvalues, while blue signifies low eigenvalues. The greater the distance of a point from the zero reference SHAP value, the more significant its impact on the output. In Figure 4B, the SHAP dependence plot illustrates the SHAP values for the POSSUM Physiology Score across all samples, with each point representing an individual sample. From the graph, it can be preliminarily deduced that the POSSUM Physiology Score is positively correlated with the SHAP value. That is, under stable conditions for other features, an increase in the POSSUM Physiology Score corresponds to a larger SHAP value and a higher final prediction probability. In Figure 4C, the SHAP dependence plot illustrates the SHAP values for the ASA classification across all samples. The ASA classification demonstrates a positive correlation with the SHAP value. Specifically, under stable conditions for other features, an increase in the ASA classification corresponds to a larger SHAP value and, accordingly, a higher final prediction probability.

In Figure 5A, the SHAP decision plot of a correctly classified case. At the bottom of the SHAP decision plot lies the baseline value. From the bottom to the top, the contribution of each feature is visualized, with the connecting line denoting the predictive process from the baseline to the ultimate outcome. In Figure 5B, SHAP decision plot of an incorrectly classified case. In Figure 5C, the SHAP force plot of a correctly classified case. The SHAP value denotes the predictive associated features of an individual patient and the respective contributions of each feature to outcome prediction. The prominent numbers represent the probability-predicted values, while the underlying values are the predicted outcomes that have not been incorporated into the model. The red element (on the left) signifies the factor that augments risk, whereas the blue element (on the right) indicates the factor that mitigates risk. The length of the arrow reflects the extent of influence on the prediction, i.e., the longer the arrow, the more pronounced the effect. In Figure 5D, a SHAP force plot of an incorrectly classified case is given.

### Model Visualization

**Figure 6:**
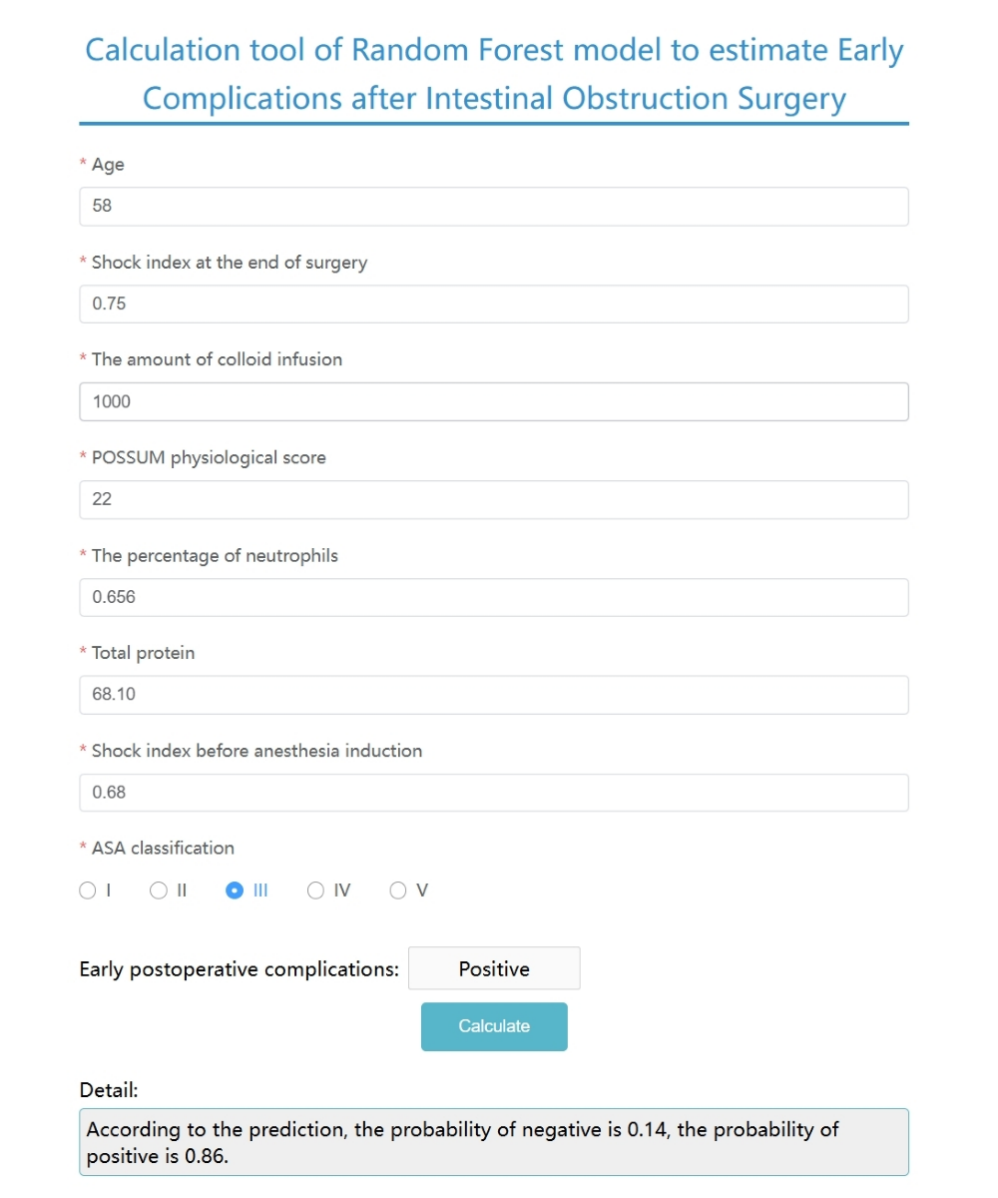
Calculation tool of random forest model to estimate early complications after intestinal obstruction surgery. After inputting 8 variables into the random forest model, the prediction model output for the case was “positive” with a probability of 86%.

We visualized the RF model and created a web-based online risk calculator [17]. The Python code of this online risk calculator is mentioned in Multimedia Appendix 2. By inputting the variables required for the RF model, the incidence of early postoperative complications for a certain patient can be predicted. As shown in Figure 6, after inputting the patient’s age of 58 years, SI of 0.75 at the end of surgery, intraoperative colloid infusion volume of 1000 mL, POSSUM physiological score of 22, neutrophil percentage of 0.656, preoperative total protein of 68.10 g/L, SI of 0.68 before anesthesia induction, and ASA classification III, the probability of early postoperative complications for this patient was found to be 86%.

## Discussion

### Principal Findings

Precisely forecasting the occurrence of postoperative complications in intestinal obstruction is of great significance for enhancing perioperative decision-making. The purpose of this study is to assess the risk of complications after intestinal obstruction surgery and provide an in-depth interpretation of the model’s decisions and their impact on variables using ML algorithms. Finally, we transformed the optimal model into a web-based risk calculator to assist clinicians in identifying high-risk patients. To the best of our knowledge, this is the first study to predict postoperative complications of intestinal obstruction surgery based on the ML algorithm. The findings show that compared with other testing algorithms, RF outperforms the strongest performance, with AUROC, specificity, sensitivity, and accuracy of 0.788, 0.810, 0.695, and 0.756, respectively. Compared with the AGESS-SBO score, the RF model achieved a better AUROC of 0.912 in predicting postoperative complications of patients with SBO. This attributed to, on the one hand, our study cohort comprised solely of patients undergoing surgical intervention; on the other hand, the ML model incorporated some intraoperative variables, making it more suitable for patients undergoing surgical treatment.

Intestinal obstruction is frequently accompanied by symptoms such as fever, disruption of the internal environment, infection, poisoning, and shock [18], which can further exacerbate the condition in the context of surgery and anesthesia. Consequently, these patients often experience various complications postoperatively. However, there is a scarcity of reliable statistical data available to demonstrate the severity and harmfulness of these complications. Therefore, we first performed a comprehensive statistical analysis and classification. First, nearly half of the patients with intestinal obstruction had early postoperative complications, causing increased hospitalization costs, prolonged hospital stays, a huge medical burden, reduced functional abilities, and even death. The most common complication was an infection because in this kind of patient, it is easy to lose the barrier effect of the intestinal mucosa and wall on bacteria, leading to bacterial translocation [19]. Second, we used the Clavien-Dindo classification to demonstrate the distribution of different degrees of complications. To improve postoperative quality of life, the emergence of disease prediction models may provide a solution for the prevention of early postoperative complications.

In the real world, clinically generated data depend on hospitals and are diversely distributed. Therefore, it is difficult to develop a robust prediction model suitable for multiple institutions or even regions. However, this study showed that with a large amount of data available, a more robust model can be developed with only a few variables. Therefore, we selected many perioperative features to find new associations in the complex relationship between a large data volume and outcome. However, the substantial number of variables within a small cohort with limited events escalates the risk of errors, including overfitting, bias or variance, and data imbalance. To minimize these errors, we have adopted the following methods: (1) performed LASSO regression analysis for variable selection to decrease the number of variables processed by the model, thereby reducing feature dimensions and mitigating the risk of overfitting; (2) preprocessed the data, including filling in missing values and standardization, to ensure effective learning by the model; (3) used cross-validation techniques to assess model performance, which aids in detecting bias or variance issues and provides insights for model improvement; (4) used data from various regions and hospitals across different time periods for external validation to confirm its applicability across different cohorts and test for overfitting; (5) used ensemble learning techniques (RF) to reduce the risk of overfitting; (6) decreased the number of samples with majority negative outcomes to amplify the signal of minority positive outcomes, thereby balancing outcome distribution and enhancing model performance; and (7) selected multiple appropriate evaluation indicators to comprehensively evaluate its performance, such as AUROC, sensitivity, accuracy, *F*_1_-score value, and so on.

Understanding how the RF model prioritizes specific features for prediction is essential to build trust among clinicians and integrate this tool into routine practice. RF is an ensemble learning method that combines multiple decision trees to improve the accuracy and robustness of predictions. Each tree in the forest is built from a random sample of the data with replacement, known as bootstrap sampling. At each node of the tree, only a random subset of features is considered for splitting, which helps prevent overfitting and increases diversity among the trees. The final prediction is made by aggregating the results of all trees. Moreover, RF provides a measure of feature importance by evaluating how much each feature contributes to decreasing impurity across all trees. Features causing significant decreases in impurity are considered more important.

The optimization of some potential intervenable variables is highly important in preventing early postoperative complications, such as SI. SI has more advantages than other vital signs in evaluating systemic perfusion, the timing of vasopressin selection, and prognosis [20,21]. Several studies [22,23] found that the incidence of hypotension in patients with a high SI increased after airway establishment. A larger amount of colloid infusion was associated with a higher incidence of early postoperative complications. On the one hand, patients with intestinal obstruction have a high probability of sepsis. Studies have confirmed that colloidal solution is not beneficial to sepsis and even increases the risk of acute renal injury [24]. On the other hand, severely insufficient blood volume during surgery often requires the infusion of more colloids. Several studies have also shown that ASA classification [3,25], age [26], and POSSUM physiological score [6,27] are independent risk factors for early postoperative complications. They have been widely applied to evaluate surgical tolerance and risk stratification. These findings are also supported by the results of this study. The percentage of neutrophils and total protein were found in a previous study to predict the occurrence of intestinal obstruction [28]. Our results indicate that they are sensitive biomarkers for predicting early postoperative complications of intestinal obstruction. Hematological parameters and markers have been widely used to predict the risk of postoperative complications [29,30].

The complex and diverse perioperative pathophysiology of patients with intestinal obstruction poses significant challenges for clinicians. Our constructed online risk calculator based on the RF model aims to facilitate timely decision-making and intervention in disease progression. However, the AUROC of the ML model seldom surpasses 0.8. This might be attributed to the fact that postoperative complications represent a mixed and complex diagnosis, with different complications potentially having specific predictive factors. Consequently, achieving higher performance indicators may be infeasible. Nonetheless, external validation results indicating a higher AUROC demonstrate the model’s generalization capability. The inclusion of higher-dimensional information, such as image or video data, in the prediction of complications may potentially enhance the predictive abilities of the model.

In clinical practice, the acceptability and applicability of a scoring system or model are determined not only by its accuracy but also by its simplicity and universality. The online risk calculator, constructed using ML algorithms, possesses these characteristics. First, this online risk calculator incorporates only 8 features, which do not augment the workload or memory burden for clinicians. Second, the calculator’s result requirements can be fulfilled during the routine clinical diagnosis and treatment process, obviating the need for additional tests or examinations and thereby avoiding any additional burden on patients. Finally, our findings indicate that the online risk calculator based on the RF model exhibits generalizability and demonstrates strong predictive capabilities across diverse regions, hospitals, and time periods. Facilitating clinicians’ decision-making processes and enhancing patient prognoses at nearly zero cost is an essential value of a scoring system. This online risk calculator can help anesthesiologists predict patients’ prognoses through partial preoperative and intraoperative information and increase clinical vigilance to make early interventions.

There are still several limitations of our study. First, it is a small sample study, and the predictive model requires a larger sample for further development and verification. Second, the current study is a retrospective study, in which there may be some confounding factors. In addition, the demographic diversity and clinical settings of the development set and validation set were not compared in this study. Future research needs to further compare more characteristics of the development and validation sets to clarify the “Reproducibility” or “Transportability” of the model. Furthermore, the model cannot be used for risk prediction of specific complications. In the future, we will construct a series of submodels for predicting the risk of specific postoperative complications in patients with intestinal obstruction. Finally, our research focused on early complications that may prolong the length of hospitalization and rehabilitation of patients after hospitalization, so there was no survival analysis. We will further explore them in the future.

### Conclusions

We constructed an online risk calculator based on the RF model, which includes 8 variables, to assist clinicians in identifying high-risk patients after intestinal obstruction surgery.

## Appendix

Multimedia Appendix 1 Additional online content.

Multimedia Appendix 2 The Python code used in the online risk calculator.

## Abbreviations

AAST: American Association for the Surgery of Trauma
AGESS-SBO: Acute General Emergency Surgical Severity–Small Bowel Obstruction
ASA: American Society of Anesthesiologists
ASN: Association of Surgeons of the Netherlands
AUROC: area under the receiver operating characteristic curve
ICD-10: International Statistical Classification of Diseases and Related Health Problems 10th Revision
INSPIRE: Informative Surgical Patient dataset for Innovative Research Environment
LASSO: least absolute shrinkage and selection operator
ML: machine learning
POSSUM: Physiological Severity Score for the Enumeration of Mortality and Morbidity
RF: random forest
ROC: receiver operating characteristic
SBO: small-bowel obstruction
SHAP: Shapley Additive Explanations
SI: shock index

## Data Availability

The datasets generated or analyzed during this study are available from the corresponding author on reasonable request.
