# Supplementary material for: Machine Learning–Based Prediction of Early Complications Following Surgery for Intestinal Obstruction: Multicenter Retrospective Study
Source: J Med Internet Res. 2025 Mar 3;27:e68354. doi: 10.2196/68354 (PMC11914837; doi:10.2196/68354)
Supplement: Multimedia Appendix 1 [file jmir_v27i1e68354_app1.docx]

**Table S1.** Clavien-Dindo classification criteria.

**Grade Definition**

Grade I Any deviation from the normal postoperative course without the need for pharmacological treatment or surgical, endoscopic, and radiological interventions

Allowed therapeutic regimens are: drugs as antiemetics, antipyretics, analgetics, diuretics, electrolytes, and physiotherapy. This grade also includes wound infections opened at the bedside

Grade II Requiring pharmacological treatment with drugs other than such allowed for grade I complications Blood transfusions and total parenteral nutrition are also included

Grade III Requiring surgical, endoscopic or radiological intervention

Grade IIIa Intervention not under general anesthesia

Grade IIIb Intervention under general anesthesia

Grade IV Life-threatening complication (including CNS complication)*requiring IC/ICU management

Grade IVa Single organ dysfunction (including dialysis)

Grade IVb Multiorgan dysfunction

Grade V Death of a patient

Suffix “d” If the patient suffers from a complication at the time of discharge , the suffix “d” (for “disability”) is added to the respective grade of complication. This label indicates the need for a follow-up to fully evaluate the complication.

*Brain hemorrhage, ischemic stroke, subarrachnoidal bleeding, but excluding transient ischemic attacks.

**Reference**

1. Dindo D, Demartines N, Clavien PA. Classification of surgical complications: a new proposal with evaluation in a cohort of 6336 patients and results of a survey. Ann Surg. 2004 Aug;240(2):205-13.

**Table S2.** Characteristics of non-postoperative complication and postoperative complication group in development cohort.

| **Variables** | **Non-complication (n=195)** | **Complication (n=176)** | **P_value** |
| --- | --- | --- | --- |
| **Basic variables** |  |  |  |
| Age (y) | 57.00 (41.50, 66.50) | 63.50(51.00, 74.00) | <0.001 |
| Gender |  |  | 0.947 |
| Female | 67 (34.36%) | 62 (35.23%) |  |
| Male | 128 (65.64%) | 114 (64.77%) |  |
| Height (cm) | 165.00 (160.00, 170.00) | 163.50 (158.00, 170.00) | 0.143 |
| Weight (kg) | 56.00 (50.00, 62.00) | 56.50 (50.00, 65.00) | 0.6 |
| BMI (kg*m^-2^) | 20.94 (19.03, 23.73) | 21.16 (19.05, 24.22) | 0.904 |
| **Preoperative variables** |  |  |  |
| Hypertension | 34 (17.44%) | 49 (27.84%) | 0.023 |
| Diabetes | 21 (10.77%) | 24 (13.64%) | 0.493 |
| Arrhythmia | 16 (8.21%) | 26 (14.77%) | 0.067 |
| Cardiovascular diseases | 52 (26.67%) | 71 (40.34%) | 0.007 |
| NYHA functional class |  |  | <0.001 |
| I | 177 (90.77%) | 132 (75.00%) |  |
| II | 15 (7.69%) | 33 (18.75%) |  |
| III | 3 (1.54%) | 11 (6.3%) |  |
| Respiratory diseases | 27 (13.85%) | 52 (29.55%) | <0.001 |
| Neurological diseases | 4 (2.05%) | 9 (5.11%) | 0.187 |
| Digestive diseases | 43 (22.05%) | 33 (18.75%) | 0.511 |
| Urinary diseases | 12 (6.15%) | 12 (6.82%) | 0.961 |
| Endocrine diseases | 21 (10.77%) | 25 (14.21%) | 0.398 |
| Hematological diseases | 2 (1.03%) | 7 (3.98%) | 0.132 |
| Hepatic insufficiency | 7 (3.59%) | 11 (6.25%) | 0.343 |
| Renal insufficiency | 7 (3.59%) | 16 (9.09%) | 0.048 |
| Coronary artery disease | 7 (3.59%) | 11 (6.25%) | 0.343 |
| Smoking | 29 (14.87%) | 24 (13.64%) | 0.849 |
| Alcohol abuse | 15 (7.69%) | 10 (5.68%) | 0.573 |
| Long-term medication | 51 (26.15%) | 59 (33.52%) | 0.15 |
| Previous surgical history | 98 (50.26%) | 101 (57.39%) | 0.204 |
| Shock | 0 (0.0%) | 16 (9.09%) | <0.001 |
| Organ failure | 3 (1.54%) | 16 (9.09%) | 0.002 |
| Peritonitis | 56 (28.72%) | 90 (51.14%) | <0.001 |
| ASA classification |  |  | <0.001 |
| I | 24 (12.31%) | 6 (3.41%) |  |
| II | 119 (61.03%) | 71 (40.34%) |  |
| III | 47 (24.10%) | 81 (46.02%) |  |
| IV | 5 (2.56%) | 17 (9.66%) |  |
| V | 0 (0%) | 1 (0.57%) |  |
| POSSUM Physiology Score | 18.00 (14.50, 22.00) | 22.00 (16.00, 27.00) | <0.001 |
| POSSUM Surgical Score | 16.00 (13.00, 20.00) | 17.00 (15.00, 21.00) | 0.001 |
| Age adjusted Charlson comorbidity index | 5.00 (2.00, 7.00) | 6.00 (3.75, 8.00) | 0.002 |
| **Preoperative treatment** |  |  |  |
| Crystalloid(ml) | 3500.00 (1000.00, 8910.00) | 3385.00 (500.00, 10020.00) | 0.894 |
| Colloid(ml) | 0.00 (0.00, 0.00) | 0.00 (0.00, 0.00) | 0.292 |
| Blood(ml) | 0.00 (0.00, 0.00) | 0.00 (0.00, 0.00) | 0.003 |
| Total fluid infusion(ml) | 3500.00 (525.00, 8815.00) | 3460.00 (687.50, 10020.00) | 0.863 |
| Gastrointestinal decompression | 84 (43.08%) | 78 (44.32%) | 0.892 |
| Antibiotics | 150 (76.92%) | 151 (85.795%) | 0.041 |
| **Laboratory results** |  |  |  |
| WBC (*10^12^L^-1^) | 7.83 (5.69, 11.56) | 9.84 (6.61, 12.57) | 0.007 |
| HGB (g*L^-1^) | 123.00 (105.50, 137.00) | 115.00 (93.00, 132.25) | 0.005 |
| Hct (%) | 0.37 (0.06) | 0.35 (0.07) | 0.004 |
| RBC (*10^12*^L^-1^) | 4.35 (3.95, 4.75) | 4.15 (3.58, 4.65) | 0.01 |
| PLT (*10^12*^L^-1^) | 254.00 (206.50, 315.00) | 265.00 (197.25, 340.75) | 0.408 |
| NEUT% | 0.70 (0.61, 0.81) | 0.78 (0.69, 0.84) | <0.001 |
| LYMPH% | 0.19 (0.11, 0.25) | 0.14 (0.08, 0.19) | <0.001 |
| MONO% | 0.08 (0.06, 0.10) | 0.07 (0.05, 0.10) | 0.167 |
| EO% | 0.01 (0.00, 0.03) | 0.01 (0.00, 0.02) | 0.045 |
| NEUT# (*10^9*^L^-1^) | 4.90 (3.16, 8.18) | 6.24 (4.12, 9.66) | <0.001 |
| LYMPH# (*10^9*^L^-1^) | 1.20 (0.93, 1.54) | 1.08 (0.79, 1.47) | 0.02 |
| MONO# (*10^9*^L^-1^) | 0.56 (0.40, 0.82) | 0.65 (0.42, 0.83) | 0.137 |
| EO#(*10^9*^L^-1^) | 0.06 (0.02, 0.15) | 0.06 (0.01, 0.14) | 0.305 |
| BASO# (*10^9*^L^-1^) | 0.02 (0.01, 0.03) | 0.02 (0.01, 0.03) | 0.315 |
| eGFR | 79.83 (61.33, 103.76) | 75.86 (52.49, 94.61) | 0.039 |
| PH | 7.41 (7.38, 7.46) | 7.41 (7.36, 7.46) | 0.401 |
| HCO3- (mmol*L^-1^) | 22.50 (20.20, 25.10) | 22.10 (20.15, 24.90) | 0.461 |
| SBE (mmol*L^-1^) | 0.70 (-1.40, 2.10) | -0.55 (-3.10, 2.48) | 0.089 |
| K+ (mmol*L^-1^) | 3.83 (3.56, 4.15) | 3.91 (3.55, 4.36) | 0.156 |
| Na+ (mmol*L^-1^) | 138.00 (135.40, 141.00) | 137.40 (134.70, 140.00) | 0.037 |
| Ca2+ (mmol*L^-1^) | 2.24 (2.12, 2.38) | 2.19 (2.06, 2.29) | 0.003 |
| Lac (mmol*L^-1^) | 0.70 (0.50, 0.90) | 0.70 (0.60, 1.20) | 0.043 |
| CRP (mg*L^-1^) | 10.35 (4.62, 33.90) | 53.41 (10.18, 120.31) | 0.002 |
| AST (U*L^-1^) | 20.00 (16.00, 25.75) | 20.00 (16.00, 28.00) | 0.966 |
| ALT (U*L^-1^) | 14.50 (11.00, 23.00) | 14.00 (10.00, 22.00) | 0.384 |
| TP (g*L^-1^) | 63.20 (58.70, 70.25) | 61.80 (55.20, 67.40) | 0.003 |
| ALB (g*L^-1^) | 37.8 (6.20) | 35.39 (6.13) | <0.001 |
| GP (g*L^-1^) | 25.25 (21.95, 28.55) | 25.20 (21.70, 28.20) | 0.699 |
| TBIL (umol*L^-1^) | 10.33 (6.82, 14.60) | 9.90 (7.20, 15.55) | 0.782 |
| DBIL (umol*L^-1^) | 3.80 (2.40, 5.97) | 4.17 (2.57, 6.73) | 0.199 |
| IBIL (umol*L^-1^) | 6.10 (4.03, 8.60) | 5.35 (4.10, 8.38) | 0.357 |
| BUN (umol*L^-1^) | 5.03 (3.58, 6.81) | 6.34 (4.06, 8.75) | <0.001 |
| SCr (umol*L^-1^) | 68.00 (59.00, 84.15) | 71.00 (54.15, 92.00) | 0.43 |
| PT (sec) | 13.90 (13.20, 14.60) | 14.20 (13.60, 15.17) | 0.001 |
| PTA (%) | 91.00 (81.00, 98.00) | 85.50 (74.00, 94.00) | <0.001 |
| INR | 1.06 (1.01, 1.14) | 1.10 (1.04, 1.21) | <0.001 |
| Fib g/L | 3.75 (3.09, 4.59) | 4.06 (3.31, 4.97) | 0.04 |
| APTT (sec) | 37.80 (34.90, 40.60) | 37.90 (35.12, 41.38) | 0.375 |
| TT (sec) | 16.40 (15.60, 17.10) | 16.00 (15.30, 16.90) | 0.056 |
| **Disease characteristics** |  |  |  |
| Obstruction degree |  |  | 0.202 |
| Incomplete | 159 (81.54%) | 133 (75.57%) |  |
| Complete | 36 (18.46%) | 43 (24.43%) |  |
| Obstruction cause |  |  | 0.182 |
| Mechanical | 188 (96.41%) | 166 (94.32%) |  |
| Dynamic | 7 (3.59%) | 7 (3.98%) |  |
| Hematogenous | 0 (0.00%) | 3 (1.71%) |  |
| Obstruction site |  |  | 0.753 |
| Colon and rectum | 98 (50.26%) | 91 (51.71%) |  |
| Ileum | 63 (32.31%) | 59 (33.52%) |  |
| Jejunum | 22 (11.28%) | 14 (7.96%) |  |
| Multiple site | 12 (6.15%) | 12 (6.82%) |  |
| Obstructive mechanism |  |  | 0.123 |
| Block | 119 (61.03%) | 112 (63.64%) |  |
| Incarceration | 18 (9.23%) | 10 (5.68%) |  |
| Reverse | 9 (4.62%) | 7 (3.98%) |  |
| Adhesion | 35 (17.95%) | 25 (14.21%) |  |
| Paralysis | 6 (3.08%) | 5 (2.84%) |  |
| Other | 0 (0.00%) | 6 (3.41%) |  |
| Combined mechanisms | 8 (4.10%) | 11 (6.25%) |  |
| Intestinal state |  |  | 0.028 |
| survival | 155 (79.49%) | 120 (68.18%) |  |
| Hyperemia or ischemia | 18 (9.23%) | 16 (9.09%) |  |
| Necrosis | 12 (6.15%) | 19 (10.795%) |  |
| Perforation | 10 (5.13%) | 21 (11.93%) |  |
| Days from onset to admission (days) | 5.00 (2.00, 10.00) | 5.00 (2.00, 10.00) | 0.283 |
| Days from admission to operation (days) | 5.00 (1.00, 10.00) | 3.00 (0.00, 8.00) | 0.108 |
| Total morbidity days (days) | 12.00 (5.00, 19.50) | 9.50 (4.00, 20.00) | 0.173 |
| **Intraoperative variables** |  |  |  |
| Operation |  |  | <0.001 |
| Open | 119 (61.03%) | 149 (84.66%) |  |
| Laparoscope | 61 (31.28%) | 21 (11.93%) |  |
| Laparoscopic to open | 15 (7.69%) | 6 (3.41%) |  |
| Operation duration (h) | 168.00 (117.50, 255.00) | 197.00 (154.50, 280.00) | 0.001 |
| Type of anesthesia |  |  | 0.01 |
| General anesthesia | 168 (86.15%) | 163 (92.61%) |  |
| CSEA | 9 (4.62%) | 0 (0.00%) |  |
| Combined with nerve block anesthesia | 18 (9.23%) | 12 (6.82%) |  |
| Duration of anesthesia (h) | 238.00 (180.00, 320.00) | 270.00 (210.00, 352.50) | 0.005 |
| Prolonged hypotension | 44 (22.56%) | 74 (42.05%) | <0.001 |
| Myocardial ischemia | 7 (3.59%) | 12 (6.82%) | 0.241 |
| Arrhythmia | 144 (73.85%) | 135 (76.71%) | 0.606 |
| Hypoxemia | 6 (3.08%) | 16 (9.09%) | 0.026 |
| **Intraoperative fluid and transfusion** |  |  |  |
| Crystalloid (ml) | 1200.00 (750.00, 1700.00) | 1700.00 (1200.00, 2200.00) | <0.001 |
| Colloid (ml) | 500.00 (500.00, 500.00) | 500.00 (500.00, 1000.00) | <0.001 |
| Sodium Bicarbonate (ml) | 0.00 (0.00, 0.00) | 0.00 (0.00, 0.00) | <0.001 |
| Albumin (ml) | 0.00 (0.00, 0.00) | 0.00 (0.00, 0.00) | 0.017 |
| **Transfusion** |  |  |  |
| RBC (ml) | 0.00 (0.00, 0.00) | 0.00 (0.00, 0.00) | <0.001 |
| Plasma (ml) | 0.00 (0.00, 0.00) | 0.00 (0.00, 0.00) | 0.001 |
| Cryoprecipitate (ml) | 0.00 (0.00, 0.00) | 0.00 (0.00, 0.00) | 0.01 |
| Urine output (ml) | 475.00 (200.00, 712.50) | 500.00 (300.00, 800.00) | 0.269 |
| Blood loss (ml) | 50.00 (20.00, 100.00) | 100.00 (50.00, 200.00) | <0.001 |
| Total volume of infusion (ml) | 1800.00 (1300.00, 2512.50) | 2400.00 (1800.00, 3300.00) | <0.001 |
| Total volume of loss (ml) | 550.00 (300.00, 900.00) | 700.00 (400.00, 1050.00) | 0.019 |
| **Intraoperative medication** |  |  |  |
| Vasopressor | 66 (33.85%) | 96 (54.55%) | <0.001 |
| Norepinephrine | 49 (25.13%) | 80 (45.46%) | <0.001 |
| Duration of norepinephrine maintenance (h) | 0.00 (0.00, 0.17) | 0.00 (0.00, 3.00) | <0.001 |
| Adrenaline | 8 (4.103%) | 19 (10.795%) | 0.023 |
| Duration of adrenaline maintenance (h) | 0.00 (0.00, 0.00) | 0.00 (0.00, 0.00) | 0.014 |
| Dopamine | 18 (9.23%) | 32 (18.18%) | 0.018 |
| Duration of dopamine maintenance (h) | 0.00 (0.00, 0.00) | 0.00 (0.00, 0.00) | 0.006 |
| Nitroglycerin | 20 (10.26%) | 36 (20.46%) | 0.009 |
| Duration of nitroglycerin maintenance (h) | 0.00 (0.00, 0.00) | 0.00 (0.00, 0.00) | 0.008 |
| Urapidil | 12 (6.15%) | 8 (4.55%) | 0.649 |
| Total urapidil use (mg) | 0.0(0.0) | 0.0(0.0) | 0.472 |
| Deoxyepinephrine | 7(3.59%) | 7(3.977%) | 1 |
| Total deoxyepinephrine use (ug) | 0.00 (0.00, 0.00) | 0.00 (0.00, 0.00) | 0.824 |
| Dexmedetomidine | 111 (56.92%) | 96 (54.55%) | 0.722 |
| Duration of dexmedetomidine maintenance (h) | 0.80 (0.00, 2.06) | 0.73 (0.00, 2.52) | 0.77 |
| Furosemide | 16 (8.21%) | 42 (23.86%) | <0.001 |
| the dose of furosemide (mg) | 0.00 (0.00, 0.00) | 0.00 (0.00, 0.00) | <0.001 |
| sodium phosphocreatine | 8 (4.10%) | 21 (11.93%) | 0.009 |
| Hormone |  |  | 0.001 |
| No | 154 (78.97%) | 114 (64.77%) |  |
| Methylprednisolone | 22 (11.28%) | 48 (27.27%) |  |
| Dexamethasone | 18 (9.23%) | 12 (6.82%) |  |
| Methylprednisolone combined with dexamethasone | 1 (0.51%) | 2 (1.14%) |  |
| Kcl (mg) | 0.00 (0.00, 0.00) | 0.00 (0.00, 0.00) | 0.294 |
| Cacl2 (mg) | 0.00 (0.00, 0.00) | 0.00 (0.00, 0.00) | 0.015 |
| **Shock index** |  |  |  |
| When entering the operating room | 0.58 (0.49, 0.68) | 0.67 (0.54, 0.78) | <0.001 |
| Before anesthesia induction | 0.59 (0.50, 0.70) | 0.68 (0.55, 0.79) | <0.001 |
| After anesthesia induction | 0.71 (0.60, 0.84) | 0.76 (0.61, 0.94) | 0.053 |
| At the end of the surgery | 0.60 (0.51, 0.72) | 0.68 (0.59, 0.81) | <0.001 |
| **postoperative features** |  |  |  |
| ICU length of stay (h) | 0.00 (0.00, 0.00) | 0.00 (0.00, 43.00) | ＜0.001 |
| Total hospitalization days(d) | 14.00 (10.00, 19.00) | 21.00 (13.00, 30.25) | ＜0.001 |
| total hospitalization cost (yuan) | 59089.71 (36201.20, 74750.44) | 85368.53 (64890.93, 116903.62) | ＜0.001 |
| postoperative hospitalization days (d) | 8.00 (7.00, 10.00) | 14.00 (10.00, 23.00) | ＜0.001 |
| Time of first postoperative defecation (d) | 3.00 (2.00, 5.00) | 4.00 (3.00, 6.00) | ＜0.001 |
| time of drainage tube retention (d) | 4.00 (1.00, 6.00) | 6.00 (3.00, 8.00) | ＜0.001 |

Note: Classification variables are expressed as cases (percentage). Continuous variables are expressed as mean (standard deviation) for normal distribution or median (interquartile range) for non-normal distribution. A total of 127 items were included in the single factor analysis. The comparison between the two groups was statistically significant (p<0.05). There were 71 data items, including 37 preoperative variables and 34 intraoperative variables.

Abbreviations: BMI, body mass index; WBC: white blood cell; HGB: Hct: Hematocrit; RBC: red blood cell; PLT: platelets; NEUT%: percentage of neutrophils; LYMPH%: percentage of lymphocytes; MONO%: percentage of monocytes; EO%: percentage of eosinophils; NEUT#: neutrophils count; LYMPH#: lymphocytes count; MONO#: monocytes count; EO#: eosinophils count; BASO#: basophil count; eGFR: estimated glomerular filtration rate; SBE: standard base excess; K+: serum potassium; Ca2+: serum calcium; Lac: lactic acid; CRP: C-reactive protein; AST: Aspartate aminotransferase; ALT: alanine aminotransferase; TP: total protein; ALB: albumin; GP: globulin; TBIL: total bilirubin; DBIL: direct bilirubin; IBIL: indirect bilirubin; BUN: blood urea nitrogen; SCr: serum creatinine; PT: prothrombin time; PTA: prothrombin time activator; INR: international normalized ratio; Fib: fibrinogen; APTT: activated partial thromboplastin time; TT: Thrombin Time; CSEA: combined spinal-epidural anesthesia.


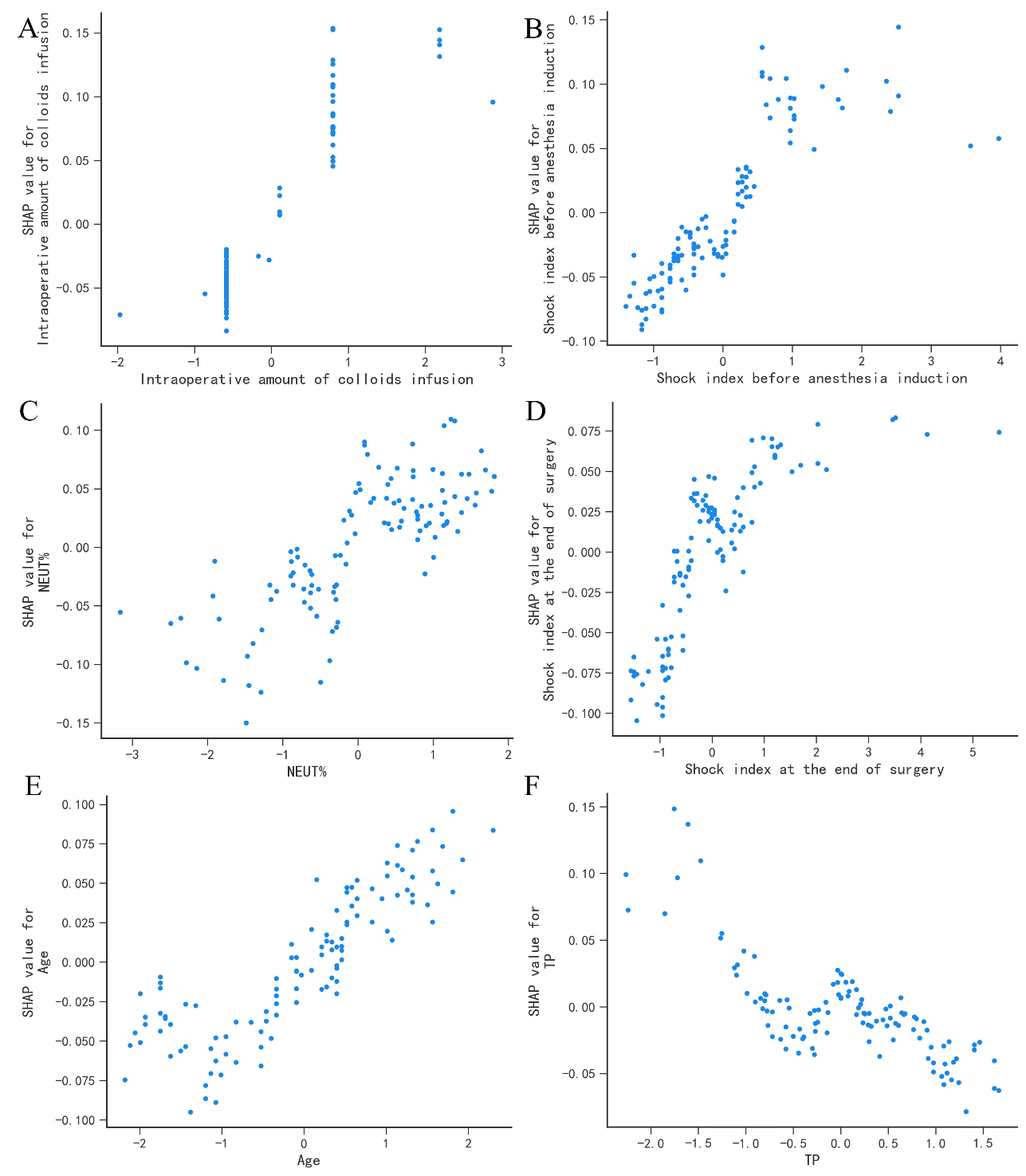


**Figure S1.** Use SHAP dependence plot to demonstrate the importance and impact of variables on the RF model. **A**: The SHAP dependence plot illustrates the SHAP values for the intraoperative amount of colloid infusion; B: The SHAP dependence plot illustrates the SHAP values for shock index before anesthesia induction; C: The SHAP dependence plot illustrates the SHAP values for the percentage of neutrophils; D: The SHAP dependence plot illustrates the SHAP values for shock index at the end of the surgery; E: The SHAP dependence plot illustrates the SHAP values for age; F: The SHAP dependence plot illustrates the SHAP values for the total protein.
